# Students’ intentions to practice primary care are associated with their motives to become doctors: a longitudinal study

**DOI:** 10.1186/s12909-021-03091-y

**Published:** 2022-01-11

**Authors:** Eva Pfarrwaller, Lionel Voirol, Giovanni Piumatti, Mucyo Karemera, Johanna Sommer, Margaret W. Gerbase, Stéphane Guerrier, Anne Baroffio

**Affiliations:** 1 University Institute for Primary Care (IuMFE), Faculty of Medicine, University of Geneva, Rue Michel-Servet 1, 1211 Genève 4, Switzerland; 2 Unit of Development and Research in Medical Education, Faculty of Medicine, University of Geneva, Geneva, Switzerland; 3 Research Center for Statistics, Geneva School of Economics and Management, University of Geneva, Geneva, Switzerland; 4 Institute of Public Health, Faculty of BioMedical Sciences, Università della Svizzera Italiana, Lugano, Switzerland; 5 Faculty of Science, University of Geneva, Geneva, Switzerland

**Keywords:** Career choice, Primary care, Undergraduate medical education, Cohort study

## Abstract

**Background:**

Medical schools can contribute to the insufficient primary care physician workforce by influencing students’ career preferences. Primary care career choice evolves between matriculation and graduation and is influenced by several individual and contextual factors. This study explored the longitudinal dynamics of primary care career intentions and the association of students’ motives for becoming doctors with these intentions in a cohort of undergraduate medical students followed over a four-year period.

**Methods:**

The sample consisted of medical students from two classes recruited into a cohort study during their first academic year, and who completed a yearly survey over a four-year period from their third (end of pre-clinical curriculum) to their sixth (before graduation) academic year. Main outcome measures were students’ motives for becoming doctors (ten motives rated on a 6-point scale) and career intentions (categorized into primary care, non-primary care, and undecided). Population-level flows of career intentions were investigated descriptively. Changes in the rating of motives over time were analyzed using Wilcoxon tests. Two generalized linear mixed models were used to estimate which motives were associated with primary care career intentions.

**Results:**

The sample included 217 students (60% females). Career intentions mainly evolved during clinical training, with smaller changes at the end of pre-clinical training. The proportion of students intending to practice primary care increased over time from 12.8% (year 3) to 24% (year 6). *Caring for patients* was the most highly rated motive for becoming a doctor*.* The importance of the motives *cure diseases*, *saving lives*, and *vocation* decreased over time. Primary care career intentions were positively associated with the motives *altruism* and *private practice*, and negatively associated with the motives *prestige*, *academic interest* and *cure diseases*.

**Conclusion:**

Our study indicates that career intentions are not fixed and change mainly during clinical training, supporting the influence of clinical experiences on career-related choices. The impact of students’ motives on primary care career choice suggests strategies to increase the attractivity of this career, such as reinforcing students’ altruistic values and increasing the academic recognition of primary care.

**Supplementary Information:**

The online version contains supplementary material available at 10.1186/s12909-021-03091-y.

## Background

Medical schools’ role in students’ career choices has become a research topic of interest in the last decades, especially considering the international consensus that medical schools should be accountable towards society [1]. The field of primary care is still struggling with workforce shortages worldwide, and many studies have tried to contribute to finding responses to the recruitment problem. Undergraduate medical education has been recognized as one of the most important influences on students’ attitudes towards primary care careers [2]. Therefore, strategies to promote primary care to medical students would benefit from a better understanding of how career aspirations develop over time [3–5]. This article focuses on the longitudinal dynamics of primary care career intentions in a student cohort and explores how they relate to students’ overall motivation to become doctors.

### Definition of primary care

The term “primary care” refers to the provision of integrated, accessible health care services to the whole population, serving as first point of contact in the healthcare system and addressing most health care needs [6, 7]. Although this definition is universal, the type of physicians practicing primary care (in terms of postgraduate specialization) differs between countries. In the United States, for example, a primary care physician (PCPs) is a specialist in family medicine, general internal medicine or general pediatrics who provides primary care services [7]. In several European countries, there is a specific specialization for PCPs, such as *general practice* in the United Kingdom or *médecine générale* in France. In Switzerland, there is no specific primary care specialization: general internists and pediatricians working in private practice are considered PCPs [8].

### The role of medical schools in promoting primary care careers

The beneficial impact of a strong primary care base on population health is well known: It decreases overall and disease-specific mortality, improves quality of care, and makes access to health care more equitable [9]. However, in many countries, the number of young physicians choosing to practice primary care is insufficient to replace the ageing workforce and to respond to population health needs [10]. Although multiple factors need to be tackled to increase the attractivity of primary care [8, 11], medical schools are an important part of the puzzle. Several systematic reviews have suggested ways in which medical schools may encourage students to pursue a postgraduate career in primary care but have also highlighted that the multifactorial aspects of career choice need to be considered more. For example, introducing a primary care clerkship without other measures does not seem to be sufficient to increase the number of students pursuing a primary care career [2, 4, 12]. Also, career choice is a longitudinal process: students spend several years in undergraduate education, during which they are not only subject to a number of educational experiences, but also evolve on a personal level, for example through socialization and by developing their professional identity [13]. In a recently published conceptual framework of medical students’ primary care career choice, based on previously published models and theories, and inspired by a systematic review, we attempted to represent the interplay of these various elements [3]. The framework represents career choice as a dynamic concept, suggesting that students’ career intentions change over the course of medical school, moving between primary care, non-primary care, and undecided. These changes may be due to varying external influences (such as moving from a more theory-based curriculum in earlier years to more practice-based teaching in later years) and evolving students’ characteristics (such as personal values or motivational factors).

### Previous research on primary care career choice

Previous research has identified several individual and contextual factors associated with medical students’ career choice [5]. Primary care career choice is related to students’ demographic characteristics (e.g., age, gender) [14], students’ professional needs to be satisfied (e.g., varied scope of practice, patient contact, income expectations, prestige, or academic opportunities) [15–18], the medical curriculum [12], as well as medical school characteristics (e.g., medical school culture) [2]. The factors determining career preferences may change during medical studies, moving from more intrinsic (e.g., self-confidence or positive attitude towards patients) to more extrinsic factors (e.g., status, workload, personal experiences in a specific specialty) [5]. The career choice process may also be influenced by students’ motives to become doctors. Among these motives, altruistic reasons (e.g., helping people) and scientific interest have been underlined as the most important drivers, followed by career- and work-related factors such as prestige, reputation, or income [19–21]. Personal characteristics, notably gender, have been found to influence students’ motives, with intrinsic motives being more important for females and extrinsic motives for males [19, 22]. Specifically, primary care career choice has been linked to motives such as interest for patient contact or low career expectations [23].

Most studies on career choice have used cross-sectional methods, and the distinction between career choice (i.e., a final decision) and career intentions (i.e., a preference which may not be final) has not always been clear [24–26]. Longitudinal studies in this field are often limited to measures at only two time points (usually at the beginning and end of medical school) and have suggested that students’ interest in primary care may decrease [25, 27] or increase over time [28]. Similarly, medical students’ motives to become doctors have mostly been explored cross-sectionally, usually at the beginning of medical school, assuming them to be stable over time. To the best of our knowledge, no study has yet specifically explored the influence of students’ general motives for becoming doctors on their subsequent specialty choice, in particular that of primary care.

### Aim and objectives of this study

Thus, although we know that students’ career intentions evolve during medical school and may be impacted by motivational factors, we still know little about when and how often these changes occur. Accordingly, longitudinal cohort studies with multiple measures have been repeatedly called for in the career choice literature [2, 4, 5, 12]. A deeper understanding about how career intentions evolve over time and their relationship with students’ characteristics could indicate strategies for medical schools to support and enhance intentions leading to primary care career choices in a comprehensive way, as has been suggested by the conceptual framework mentioned above. Thus, the aim of this study was to provide a longitudinal exploration of the association between students’ motives for becoming doctors and primary care career intentions, and how these two elements evolved over time. Our objectives were to explore, in a cohort of undergraduate medical students followed over a four-year period: (1) the longitudinal dynamics of students’ career intentions in relation to primary care; (2) the stability of students’ motives for becoming doctors over time; and (3) the relationship between students’ motives for becoming doctors and their primary care career intentions over the four-year period.

## Methods

### Educational context

The six-year undergraduate curriculum at the Faculty of Medicine in Geneva consists of a pre-selection year (year 1, at the end of which students pass a selection examination to be admitted to year 2), two pre-clinical years (years 2 and 3), two clinical years (years 4 and 5), and one elective year (year 6), integrating acquisition of theoretical knowledge and clinical competencies. Primary care is taught through theoretical lectures and three practical clerkships with PCPs (four half-days in year 2, a four-week part-time clerkship in year 4 in the context of a clinical teaching unit in primary and community care, and a one-month full-time clerkship in year 6). In Switzerland, specialization is not regulated: students are free to choose any specialty after graduation from medical school, and it is possible (and frequent) to change specialty during postgraduate education.

### Primary care context

In Switzerland, ambulatory care is mainly provided by private practice (i.e., self-employed) physicians. Both specialists and PCPs may work in private practice; there is no mandatory gatekeeper system [8].

### Participants

Medical students who had started their medical studies (first academic year) in 2011 and 2012 were proposed to enter a cohort study after they passed the selection examination at the end of the first year. From a total of 306 students in these two classes, 290 were enrolled into the cohort study (95% of all selected students). They were invited to complete a yearly paper-and-pencil survey which they filled in during the interval between lectures. Participants signed a consent form after being informed about the content of the project, their entitlements and commitments as voluntary participants, and the terms of confidentiality. They provided their unique student identification number at each data collection to allow longitudinal matching of questionnaires. Researchers could not identify students through their identification numbers to guarantee confidentiality.

For the present study, we considered data collected over a four-year period, from the 3rd (i.e., end of pre-clinical training) to the 6th year of medical school (i.e., end of clinical training). We did not use data from the 1st and 2nd year because the survey question about future specialty choice (see below) used in this analysis was only included in the survey from the 3rd academic year onwards. Also, we were mostly interested in the clinical years of medical education for this study. To ensure a reliable statistical estimation, we included only participants who had provided relevant outcome data (motives and career intention) in at least three out of these four years (*N* = 217, i.e., 75% of the 290 students enrolled into the cohort).

### Measures

All measures were collected yearly, including gender and age. A list of *motives for becoming a doctor* was presented to the students: *academic interest, prestige*, *reward, private practice*, *saving lives, caring for patients, cure diseases, vocation, mission,* and *altruism.* They were asked to rate the importance of each motive on a 6-point Likert scale (i.e., “Describe how important each of these keywords is for your choice of medicine” from 1 = *not important at all* to 6 = *very important*). The list of motives was developed based on a review of the literature [15, 29, 30] to have a wide description of different typologies of motives for choosing medicine. *Students’ career intentions* were assessed by two single-choice questions: (1) “What type of practice do you plan to exercise in the future?” (options: private practice, hospital practice, or teaching/research); (2) “If you are considering a specialization, which one?” (options: anesthesiology, general internal medicine, internal medicine subspecialty, emergency medicine, obstetrics-gynecology, pathology, pediatrics, psychiatry, radiology, surgery, academic activity, undecided, and other). The questions were developed specifically for this survey but were similar to questions previously used in other surveys in a similar context [14]. The list of possible answers was derived from the list of postgraduate specializations available in Switzerland. For the study presented here, focusing on primary care career choice, we categorized students’ responses into *primary care* (defined as a specialty choice of general internal medicine or pediatrics, combined with private practice), *non-primary care* (other combinations of practice type and specialty, including the option “other”) and *undecided* (undecided about specialty).

### Analyses

Data were preliminarily examined for accuracy of data entry. We calculated descriptive statistics for age (mean and standard deviation), gender (N and % of females), career intention categories (N and %) and the distribution of each motive (N and medians). The population-level changes between career intention categories over the four academic years were represented in a diagram. Changes in the rating of motives over the four years were analyzed by comparing the distribution in years 4, 5 and 6 with year 3, using bar plots and Wilcoxon paired tests. Gender differences in the rating of motives were analyzed with Wilcoxon tests. In both cases, the resulting *p*-values were corrected for multiple comparisons [31].

We investigated the association between motives to become a doctor and primary care career intentions using a generalized linear mixed model [32], which extends the classical generalized linear models to account for correlation between repeated observations in individual students. As the logistic regression model only applies to dichotomous outcomes, we estimated two models: Model 1 comparing primary care career intentions to non-primary care career intentions, and Model 2 comparing primary care career intentions to undecided. To ensure a reliable estimation of the parameters, we eliminated three motives from the analysis. (Gender and age were included as covariates because of their possible association with career choice reported in the literature [14]. The covariate *academic year* was also included to account for the longitudinal element (number of years in medical school). The estimated coefficients were interpreted in terms of their magnitude (absolute value), sign (positive or negative), and *p*-value. Please refer to Additional file 1 for detailed explanations about the GLMM analysis and interpretation.

All analyses were carried out with R statistical software [33], using the estimator proposed by Breslow and Clayton [34] and implemented in the package MASS [35]. *P*-values smaller than 0.05 were considered statistically significant.

## Results

Our cohort included 217 students. The average age in the third academic year was 23 years (range: 20–41 years; SD = 2.08), and 60% (*N* = 130) were female.

### Dynamics of primary care career intentions

**Fig. 1:**
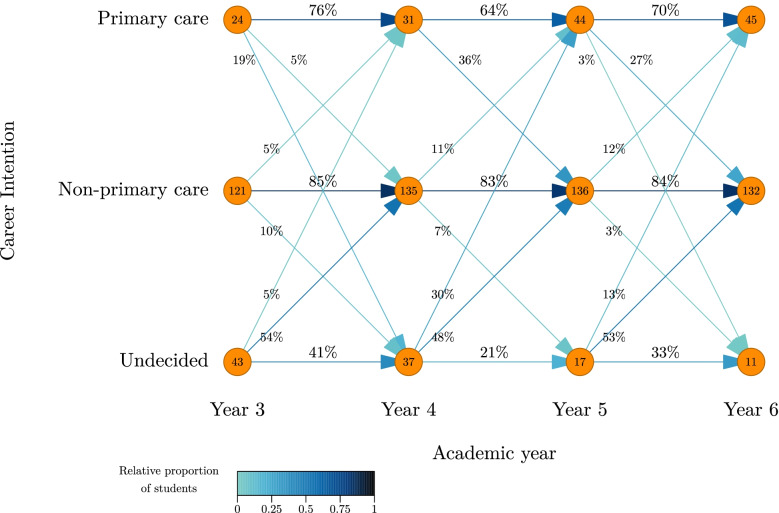
Dynamics of three categories of career intentions in a cohort of medical students. The figure represents the flows between the three career intention categories of interest over four academic years (academic year 3 to 6). Numbers in circles represent absolute numbers in each category for each academic year. Arrows represent changes between categories and academic years (percentages represent % of N in the circle from which the arrow starts)

The proportion of students intending to practice primary care steadily increased from 12.8% (*N* = 24) in year 3 to 24% (*N* = 45) in year 6. Over the same period, the proportion of undecided students gradually decreased from 22.9% (*N* = 43) to 5.9% (*N* = 11); there was a slight increase in the proportion of students intending to practice non-primary care, from 64.4% (*N* = 121) to 70.2% (*N* = 132). Figure 1 visualizes the population-wide flows between the three career intention categories over the four-year period. The greatest changes between *primary care* and *non-primary care* occurred during clinical training (i.e., between academic years 4 and 6). There was also a relatively large flow from *undecided* to *non-primary care* at the end of pre-clinical education (i.e., between academic years 3 and 4). Changes from *undecided* to *primary care* occurred predominantly between years 4 and 5.

### Stability of motives for becoming a doctor

**Fig. 2:**
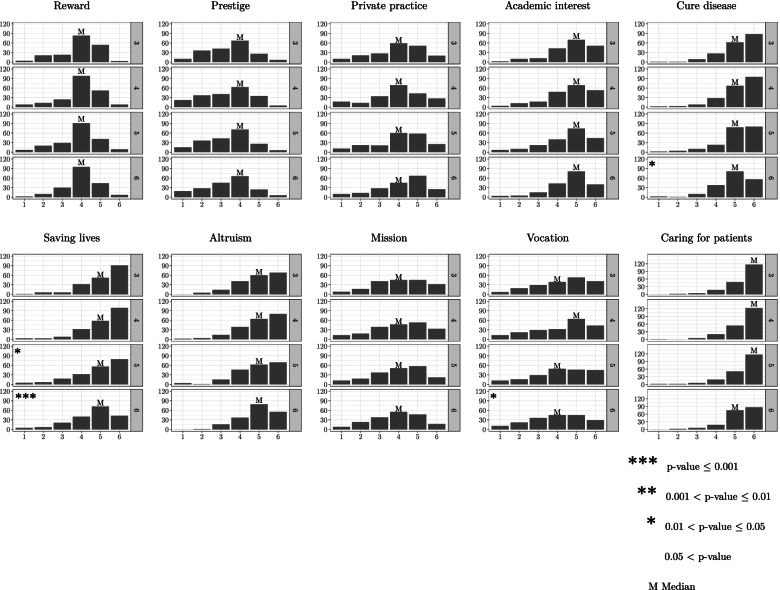
Evolution of the rating of motives for becoming a doctor. The figure represents changes in 10 motives for becoming a doctor in a cohort of 217 medical students followed over four years (academic year 3 to 6). Individual graphs represent the number of students on the y axis (on the left of the graph), separated by academic year (3 to 6: y axis on the right of each graph), and the ordinal rating of each motive on the x axis (from 1 = very little important to 6 = very important). The asterisks indicate a significant difference in the distribution of the responses of the respective year (4, 5, and 6) compared to year 3 (year of reference) for each motive

*Caring for patients* was the most highly rated motive, followed by s*aving lives, cure diseases*, *altruism,* and *academic interest* (Fig. 2). Compared to year 3, the distribution of ratings significantly decreased over time for three motives: *cure diseases, saving lives,* and *vocation*. The other motives remained stable. *Cure diseases*, *saving lives, altruism*, *mission*, and *caring for patients* were rated significantly higher by women than by men in one or several years, whereas p*restige* was rated higher by men, but only in year 3 (see Additional file 2 for results stratified by gender).

### Association between students’ motives to become doctors and their primary care career intentions

In the first model (Table 1), comparing primary care to non-primary care career intentions, we found that a higher importance rating of the motives *private practice* and *altruism* increased the probability of primary care career intentions, whereas the ratings of *prestige, academic interest* and *cure diseases* were negatively related to primary care career intentions. The absolute values of the estimated coefficients in the model suggest that *private practice* and *altruism* had greater impact on the probability of primary care career intention than *prestige* and *academic interest*. The covariate *academic year* was positively related to primary care career intentions. This means that as they advanced in medical school, students were increasingly interested in primary care as opposed to non-primary care. Finally, female gender was associated with primary care career intentions, whereas we found no significant association between age and primary care career intentions.

**Table 1:** Relation between students’ motives to become doctors and primary care versus non-primary care career intentions

Motive	Coefficient	Standard error	Degrees of freedom	t-value	***p***-value
(Intercept)	−4.724	2.686	445	− 1.759	0.079
**Private practice**	0.547	0.123	445	4.447	< 0.0001
**Altruism**	0.726	0.170	445	4.266	< 0.0001
**Academic interest**	−0.361	0.117	445	−3.074	0.002
**Prestige**	−0.381	0.124	445	−3.069	0.002
**Cure disease**	−0.359	0.176	445	−2.037	0.042
Vocation	−0.083	0.107	445	−0.769	0.442
Saving lives	0.061	0.166	445	0.367	0.714
**Academic year**	0.421	0.152	445	2.765	0.006
**Female gender**	1.127	0.465	212	2.426	0.016
Age	−0.055	0.121	445	−0.454	0.650

Estimated non-standardized coefficients from a generalized linear mixed model comparing primary care to non-primary care career intentions (model 1). Estimated variance of random effects σ ^ = 2.58

In the second model (Table 2), comparing primary care career intentions to undecided, we found that primary care career intentions were again positively associated with the motives *private practice* and *altruism* and negatively with *academic interest*. In this model, *altruism* had a particularly large effect size compared to the other motives. As in model 1, the covariate *academic year* was positively associated with primary care career intentions. There was no significant effect of gender or age.

**Table 2:** Relation between students’ motives to become doctors and primary care career intentions versus undecided

Motive	Coefficient	Standard error	Degrees of freedom	t-value	***p***-value
(Intercept)	−10.934	3.949	127	− 2.770	0.006
**Private practice**	0.610	0.208	114	2.924	0.004
**Altruism**	1.648	0.366	114	4.504	< 0.0001
**Academic interest**	−0.495	0.232	114	−2.133	0.035
Prestige	−0.101	0.232	114	−0.436	0.664
Cure disease	−0.016	0.301	114	−0.055	0.957
Vocation	−0.002	0.216	114	−0.011	0.992
Saving lives	−0.446	0.338	114	−1.320	0.189
**Academic year**	1.491	0.252	114	5.911	< 0.0001
Female gender	0.779	0.751	127	1.036	0.302
Age	−0.077	0.162	114	−0.471	0.639

Estimated non-standardized coefficients from a generalized linear mixed model comparing primary care career intentions to undecided students (model 2). Estimated variance of random effects σ ^ = 3.25

To illustrate the effect of the importance attributed to the different motives for becoming a doctor on the probability of primary care career intentions, we simulated two situations. A female student attributing high importance to prestige and academic interest, and a slightly lower importance to all other motives, would have a probability of only 3.5% to express an intention to choose primary care. This probability would however be 48% for another female student attributing high importance to private practice and altruism, and lower importance to prestige and academic interest. Additional file 1 provides more details on the interpretation of the two statistical models.

## Discussion

Our study explored the longitudinal dynamics of students’ primary care career intentions during medical school and their relationships with motives for becoming doctors. In agreement with our conceptual framework [3], our findings indicate that career choice is a dynamic process and confirm that career intentions change during the clinical training period. Most students’ motives to become doctors were stable over the years of medical education, and *altruism* and *private practice* were positively associated with primary care career intentions.

In our cohort, the proportion of students intending to become PCPs increased during undergraduate education, yet the proportion of graduating students planning to become PCPs was only about half of what has been recommended for optimal health system efficiency [36], reinforcing the need to find factors increasing the attractivity of this career. Our observational study was designed to explore changes in career intentions and motives over time, thus we cannot directly draw conclusions about what might have influenced these changes. Nevertheless, to our best knowledge, this is the first study providing an overview of the population-wide dynamics of career intentions in a student cohort with more than two longitudinal measures over time. We identified the moments when career intentions changed, thus expanding on previous evidence that career intentions change between matriculation and graduation [37]. These findings are in line with those from a recently published study of specialty preferences in a small student cohort in the Netherlands [38], which observed that career preference paths were unstable in subgroups of students. In our cohort, most changes occurred during the clinical years, suggesting that clinical experiences might have influenced career intentions [39, 40]. Notably, undecided students shifted to primary care mostly between years 4 and 5, which might be due to the primary care clerkship they attend during this period [12]. There was also a relatively important shift from *undecided* to *non-primary care* during the last part of pre-clinical education, which could be due to the relative absence of primary care in the curriculum during this period, or to the focus on theoretical teaching attracting students’ interest towards medical subspecialties. Practically, this implies that we should reflect on how to integrate primary care into pre-clinical teaching. Depending on the context, we suggest, for example, to use lectures or seminars to show the comprehensive aspect of primary care by integrating knowledge from other, more topic-specific lectures, or to use primary care settings for early clinical exposure or for practicing clinical skills. Although we cannot draw conclusions regarding the career intention dynamics of individual students, our findings suggest that career choices are formed gradually over time, sustaining the calls to longitudinally integrate primary care into the curriculum [12, 41].

Our results about how students’ motives to become doctors evolve as they advance in medical school are novel in the research literature. Overall, the most highly rated motive was *caring for patients*, followed by *saving lives*, *cure diseases* and *altruism*, supporting previous findings that students are motivated by the desire to help others and care for patients [20]. Motives linked to societal status - *prestige* and *reward* - were considered less important, confirming findings from other high-income countries [30]. Gender differences observed in our cohort confirm that women seem to be more care-oriented and men more motivated by factors such as prestige [42, 43]. However, we did not observe gender differences across all four years, suggesting that the impact of gender on students’ motives to become doctors might not be as important as previously thought. In our cohort, age was not associated with primary care career intention, which may be explained by the low variance of age across our cohort (in Switzerland, most students enter medical school directly after high school and thus have a similar age).

We evidenced a slight decrease in the importance of the motives *cure diseases, saving lives* and *vocation*. A possible explanation is that students may have a somewhat idealized image of their future profession in earlier stages, which becomes more diverse and realistic over time. Nevertheless, most motives remained stable, suggesting that they might be related to personal characteristics and are thus important factors to consider when reflecting about how to influence students’ career choices.

Primary care career intentions were positively associated with the motives of *private practice* and *altruism*. In Switzerland, PCPs work predominantly in private practice, explaining the importance of this motive, which has also been found in other countries with a similar practice context [15]. Altruistic motives have already been related to primary care career choices in previous studies [14, 22, 44], even in the context of an educational culture encouraging specialization [45]. Our findings expand on this knowledge by adding the longitudinal perspective showing that the motive of altruism was consistently associated with primary care career intentions over the four study years. This suggests that such a value could be put forward in teaching related to primary care to motivate certain groups of students (e.g., undecided students) to become PCPs.

On the other hand, three motives were negatively associated with primary care career intentions: *prestige*, *academic interest,* and *cure diseases*. Primary care is often regarded as a discipline lacking prestige and academic interest [2, 26, 46]. A specialty’s prestige is influenced by its importance in the health system and society, expressed, for example, through high income, competitiveness, or advanced technologies [47–49]. Although these aspects involve a variety of stakeholders, medical school culture is important as it determines students’ perceptions of primary care, through the representation of primary care faculty and the academic importance given to this field [2, 50]. For example, students hold negative attitudes towards primary care where primary care teaching is perceived to be of low quality [41]. On the contrary, primary care is regarded as more prestigious in contexts where students are exposed early and often to primary care and where primary care is fully recognized as an academic discipline [45]. The motive of “curing diseases” might be associated with non-primary care specialties since they are perceived as offering specific, immediate solutions to patients’ problems [51]. This would mean that primary care could potentially gain in attractiveness if the dimension of curing diseases were presented in a more comprehensive way. Overall, our findings suggest that we should reflect on how these motives could be consciously integrated into various teaching activities to give a realistic, yet attractive image of primary care. Also, qualitative research methods would be particularly appropriate to investigate the role of students’ motives in more detail.

### Strengths and limitations

Our study’s main strength is the longitudinal methodology, presenting a quantitative and dynamic picture of how career intentions evolve over a four-year period in a cohort of medical students. We also explored the relationship between primary care career intentions and students’ motives for becoming doctors over the same period. These insights may contribute valuable knowledge for ways in which medical schools can further reinforce primary care career preferences.

We acknowledge that our study has limitations. First, it took place in a single medical school with students from two classes, limiting generalizability to other contexts, as career choice is influenced by institutional factors whose impact cannot be measured in our study. Also, certain aspects related to primary care are country specific, influencing motives such as private practice. The liberal organization of postgraduate education in Switzerland makes it difficult to know when to consider a career choice to be final; we therefore only used the term “career intention” in our study, because these decisions may be revised in later years. Nevertheless, our study raises several issues related to primary care career choice that are of global interest, and we estimate that many medical schools are in a comparable context and may benefit from our findings. A further limitation is that our set of motives has not been previously validated and has not been pilot tested before use. Thus, we cannot exclude that the keywords used to describe the motives might have been diversely interpreted across students. However, it is based on a review of the literature and employs keywords that have been used in studies in similar contexts. Moreover, the coherence of ratings across years indicate that students had a consistent understanding of the motives. Also, we cannot exclude that their answers were not biased by social desirability, but as studies from other countries have generally found similar results, we consider that the overall probability of this is low. Thus, replicating our results in different contexts using the same typologies of motives for becoming doctors would be crucial, and qualitative methods could add valuable knowledge about students’ motives to become doctors and their impact on career intentions. Finally, we only analyzed population-wide changes in career intentions, highlighting *when* changes happen, but limiting conclusions about *how* they happen and about individual choice processes. Therefore, future research should aim to gain more insights into individual students’ trajectories.

## Conclusions and future directions

Our study indicates that career intentions are not fixed – on the contrary, they change over the course of the undergraduate curriculum, leaving several possibilities to enhance students’ interest in primary care. The insights about how students’ career choices are associated with their motives to become doctors can help finding ways to increase the attractivity of this career. Our findings suggest several strategies that could be implemented in undergraduate curricula: reinforcing and responding to students’ altruistic values by linking them explicitly to primary care; focusing on students attracted by private practice by integrating specific aspects into primary care teaching (such as, for example, the entrepreneurial aspects of private practice); or increasing the academic recognition of primary care to favor students’ identification with this career (for example, by making primary care research more visible and integrating students into research projects). In our future research, we plan to further investigate elements of our conceptual framework to deepen our understanding of the dynamics of individual career choice trajectories and explore the elements influencing individual students’ career intentions.

## Supplementary Information

**Additional file 1.** Details on statistical analysis. The additional file presents details of the generalized linear mixed model used to analyze the relation between motives to become a doctor and primary care career intentions and provides further explanations about the interpretation of the results of this analysis.

**Additional file 2.** Evolution of the rating of motives for becoming a doctor in a cohort of medical students followed over four years (academic year 3 to 6), stratified by gender. This supplemental figure highlights the gender differences in the rating of motives for becoming a doctor, presented by academic year.

## Data Availability

The datasets analyzed during the current study are available from the corresponding author on reasonable request.

## Abbreviation

PCP: Primary care physician
