# Supplementary material for: Students’ intentions to practice primary care are associated with their motives to become doctors: a longitudinal study
Source: BMC Med Educ. 2022 Jan 11;22:30. doi: 10.1186/s12909-021-03091-y (PMC8750802; doi:10.1186/s12909-021-03091-y)
Supplement: Supplementary file 1 — Additional file 1. Details on statistical analysis. The additional file presents details of the generalized linear mixed model used to analyze the relation between motives to become a doctor and primary care career intentions and provides further explanations about the interpretation of the results of this analysis. [file 12909_2021_3091_MOESM1_ESM.pdf]

Additional file 1: Details on statistical analysis and interpretation of results

Relation between motives to become a doctor and primary care career intentions: generalized linear mixed model

Our data are longitudinal, in the sense that we collected multiple observations of the same individual across the four years. Hence, we cannot apply a classical generalized linear model (i.e. a logistic regression in this case), since such a model would ignore the dependence between observations of the same individual. The classical approach for modelling longitudinal data of this type is to rely on generalized linear mixed models (GLMM, see below) [1], which can model the difference between groups (individuals in our case) by specifying random effects that are common to all observations of a group. Given the data provided, and as the estimation of GLMM relies on the approximation of intractable integrals and is known to be unreliable with complex random structure, we considered a simple random structure with a random intercept per individual.

GLMM are adapted for the modeling of longitudinal data where the dependent variable is assumed to follow an exponential distribution (Bernoulli in the context of this research), by allowing the inclusion and estimation of random effects in addition to fixed effects. Defining the individual $i \in \{1, \dots, N\}$ and the academic year $j \in \{3, \dots, 6\}$, and defining the number of covariates excluding the intercept $L = 10$, we define the model as:

$$ln\left(\frac{Pr\left(Y_{i,j} = 1 \vee X_{i,j}\right)}{1 - Pr\left(Y_{i,j} = 1 \vee X_{i,j}\right)}\right) = \beta_0 + S_i + \sum_{l=1}^{L} \beta_l X_{i,j,l}\, , S_i N(0, \sigma^2).$$

As the logistic regression model only allows to distinguish two classes, we first compared primary care committed students to students intending to practice a non-

primary care specialty (model 1). We then compared students intending to practice primary care to those who were undecided (model 2).

To ensure a reliable estimation of the parameters, we considered the Event Per Variable (EPV) rule of thumb (which specifies to have at least 10 observations per variable) [2] and eliminated three motives from the analysis: *reward* because of a higher number of missing data, *mission* because of its high inter-correlation with *vocation (>0.6),* and *care for patients* because of its very low variance over study years.

Interpretation and predictive power

The interpretation of our models does not differ from that of a standard logistic regression for the fixed effect. The sign of the estimated coefficients (positive or negative) indicates the direction of the effect of the variable on the probability of being assigned to the class of interest (i.e., the intention to practice primary care). In our study, a positive sign of the estimated coefficient suggests that a positive increment of the associated variable will increase the probability of being interested in a primary care career. The magnitude of the estimated coefficients is directly related to the scale of the variable. Thus, the magnitude of the estimated coefficients of two variables can only be meaningfully compared when the two variables are on the same scale. In our study, this means that the coefficients relative to the “motives” variables can be compared to each other, but that the coefficient related to the “gender” and “age” variables cannot be compared to the others, for example.

For a complete discussion of the interpretation of estimated coefficients in GLMM, we recommend [3].

The estimated variance of the random intercepts gives insights on the importance of effects that are not accounted for in our model with respect to the observed variables. We obtained a measure of the importance of the fixed effects in the modeling of the probability of primary care career intention by performing a leave-one-out cross-validation procedure (LOOCV), considering only the estimated fixed effects. We obtained a LOOCV accuracy of 78.14% (model 1) and 64.68% (model 2), suggesting that both models provide adequate predictions.

Illustration of the relationship between motives to become doctors and the probability to be interested in a primary care career

To illustrate the impact of the importance of the rating of the different motives for becoming a doctor on the probability of primary care career intentions, we created a Webapp that interested readers may access directly. By allowing to combine different ratings of motives, this app in fact visualizes the effect of the different magnitudes of the estimated coefficients of the “motives” variables (explained above) on the probability of indicating an intention to practice primary care in the future (π).

The app may be accessed here: https://data-analytics-lab.shinyapps.io/students_motives_and_PC/

References

1. Breslow NE, Clayton DG. Approximate inference in generalized linear mixed models. *J Am Stat Assoc*. 1993;88(421):9-25.

2. Peduzzi P, Concato J, Kemper E, Holford TR, Feinstein AR. A simulation study of the number of events per variable in logistic regression analysis. *J Clin Epidemiol*. 1996;49(12):1373-9.

3. Jiang J. Linear and generalized linear mixed models and their applications. Springer Science & Business Media, 2007.
