# Supplementary material for: Students’ intentions to practice primary care are associated with their motives to become doctors: a longitudinal study
Source: BMC Med Educ. 2022 Jan 11;22:30. doi: 10.1186/s12909-021-03091-y (PMC8750802; doi:10.1186/s12909-021-03091-y)

Appendix 2: Evolution of the rating of motives for becoming a doctor in a cohort of medical students followed over four years (academic year 3 to 6), stratified by gender. Students rated each motive from 1 (very little importance) to 6 (very important). Asterisks indicate a significant difference in the distribution of the responses between men and women in a given academic year.

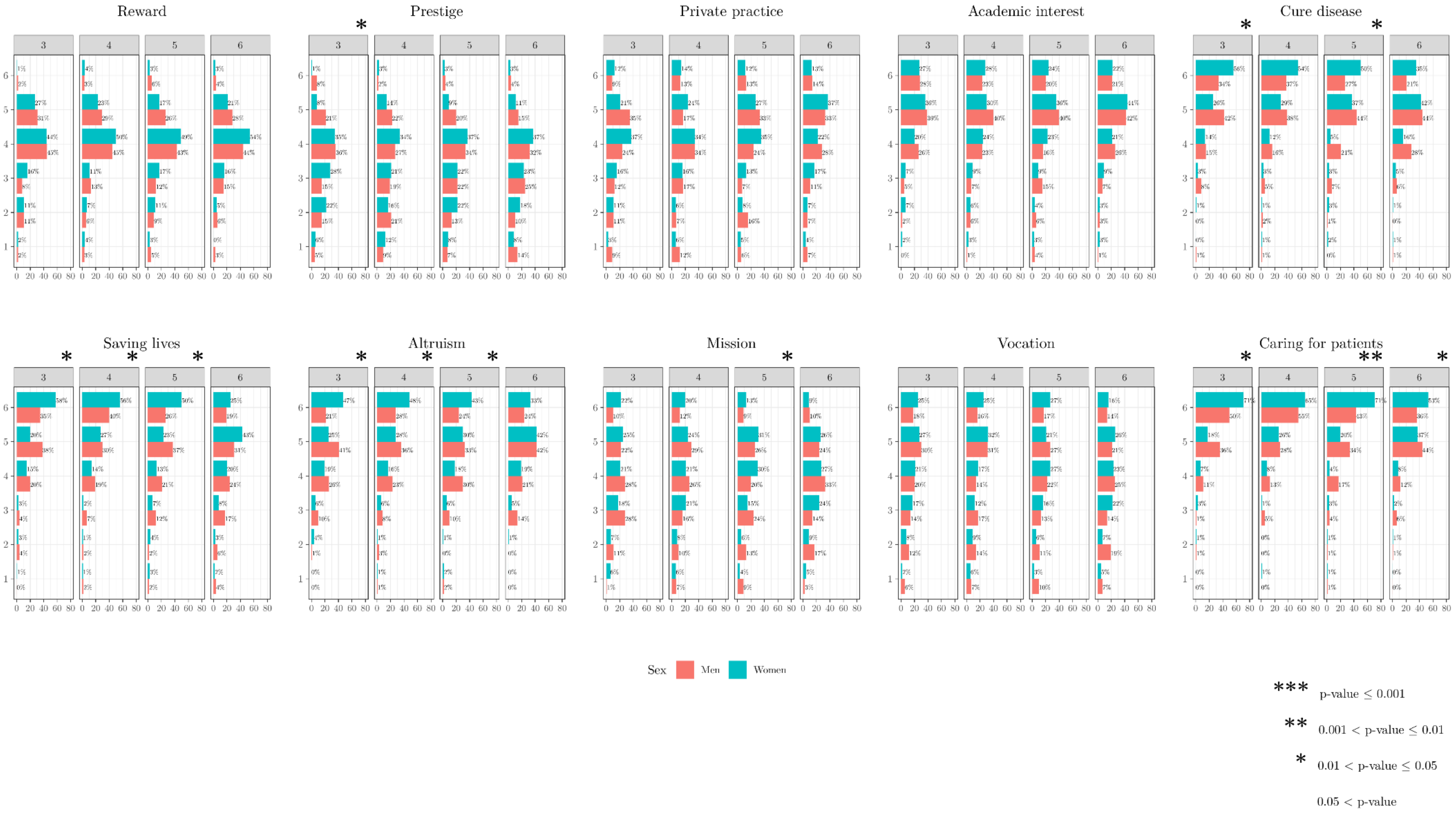

Supplement: Supplementary file 2 — Additional file 2. Evolution of the rating of motives for becoming a doctor in a cohort of medical students followed over four years (academic year 3 to 6), stratified by gender. This supplemental figure highlights the gender differences in the rating of motives for becoming a doctor, presented by academic year. [file 12909_2021_3091_MOESM2_ESM.pdf]
